# Plant-based superoxide dismutase supplementation in the athlete population

**DOI:** 10.5937/jomb0-63037

**Published:** 2026-05-22

**Authors:** Petrovičova Olina Dudašova, Brižita Đorđević, Vanja Todorović

**Affiliations:** 1 Belgrade University - Faculty of Pharmacy, Department of Bromatology, Belgrade

**Keywords:** antioxidants, athletes, exercise, oxidative stress, SOD, supplementation, antioksidansi, fizička aktivnost, oksidativni stres, SOD, sportisti, suplementacija

## Abstract

Physical exercise significantly influences the redox balance, with its intensity, duration, and type affecting the produc-tion of free radicals, including reactive oxygen and nitrogen species. An excess of free radicals can lead to oxidative stress. Elite athletes, due to rigorous training and often insufficient rest, are particularly susceptible to oxidative stress, which can negatively affect their health and sports performance. Plant-based superoxide dismutase (SOD), combined with gliadin isolated from wheat, is a relatively new dietary supplement known for its antioxidant proper-ties. The aim of this review was to evaluate the effects of SOD/gliadin supplementation among athletes. We exam-ined the impact of this supplementation on redox status, inflammatory markers, indicators of muscle damage, and sports performance. Overall, the analysis of the data indi-cated some beneficial effects on all observed aspects of athletes' health and performance. The effects of supple-mentation appear to depend on the type of sport, the ath-lete's training status, and the design of the supplementa-tion study. However, very few studies have been published on this topic; therefore, further research is needed to clarify the potential benefits of SOD/gliadin supplementation for athletes. Future studies should include a larger number of participants and implement controlled conditions with stan-dardised dosage regimens, enabling more robust, compa-rable, and reliable outcomes.

## Abstract (sr)

Fizička aktivnost ima značajan uticaj na redoks ravnotežu, pri čemu vrsta fizičke aktivnosti, njen intenzitet i dužina trajanja utiču na proizvodnju slobodnih radikala, uključujuci reaktivne vrste kiseonika i azota. Povišen nivo slobodnih radikala može dovesti do oksidativnog stresa. Elitni sportisti, usled napornih treninga i često nedovoljno odmo-ra, posebno su podložni oksidativnom stresu, što može negativno uticati na njihovo zdravlje i sportske perfor-manse. Superoksid dismutaza (SOD) biljnog porekla, kom-binovana sa glijadinom izolovanim iz pšenice, je relativno novi dijetetski suplement poznat po svojim antioksidativnim svojstvima. Cilj ovog preglednog rada bio je da se procene efekti suplementacije kombinacijom SOD/glijadin kod sportista. Ispitali smo uticaj ove suplementacije na parame-tre redoks statusa, inflamacije, oštecenja mišica i sportske performanse. Generalno, analiza podataka ukazala je na određeni nivo povoljnog uticaja na sve posmatrane para-metre zdravlja i performansi sportista. Čini se da efekti suplementacije zavise od vrste sporta, statusa utreniranosti sportiste i dizajna studije suplementacije. Međutim, objav-ljeno je veoma malo studija na ovu temu pa su potrebna dalja istraživanja kako bi se razjasnile potencijalne koristi suplementacije SOD/glijadinom u populaciji sportista. Buduće studije bi trebalo da uključe veći broj učesnika uz suplementaciju u kontrolisanim uslovima sa standardizo-vanim režimima doziranja, omogućavajući robusnije, uporedivije i pouzdanije rezultate.

## Introduction

Athletes represent a physiologically distinct population due to the intense metabolic demands of exercise. In recent decades, numerous studies following Dillard et al. [1] have confirmed that intense exercise can lead to excessive free radical production through various mechanisms. Free radicals are primarily produced during cellular respiration, particularly during the electron transfer process along the mitochondrial electron transport chain. Throughout this process, electrons are transferred to oxygen, which is then reduced to superoxide anion (O_2_'^-^), a very potent free radical [2]. Muscle cells, highly active during exercise, are a major site of this production. Additional sources include the activity of enzymes such as nicotinamide adenine dinucleotide phosphate (NADPH) oxidase, myeloperoxidase (MPO) and phospholipase A_2_-dependent processes [3][4]. Muscle energy requirements are extremely high. Adenosine triphosphate (ATP), the primary source of energy, is broken down into adenosine diphosphate (ADP) and adenosine monophosphate (AMP) to release energy and support continuous muscle contractions. The further breakdown of AMP and purines into hypoxanthine, xanthine, and uric acid includes the enzymatic activity of xanthine oxidase (XO). This form of enzyme is especially active under anaerobic conditions following intense exercise. The final electron recipient in the XO metabolic pathway is molecular oxygen, leading to O_2_'^-^ formation [4]. Exercise-induced muscle damage and subsequent phagocyte infiltration also contribute to the release of free radicals [4].

Excessive production of free radicals can lead to oxidative stress, a condition characterised by an imbalance between the generation of reactive oxygen and nitrogen species (RONS) and the body's antioxidant defences. Physical activity has a dual effect on redox balance: acute high-intensity exercise can transiently elevate oxidative stress [5][6], whereas regular moderate exercise enhances antioxidant capacity and promotes beneficial long-term adaptations [7]. Free radicals, which are highly reactive molecules, can harm lipids, proteins, and DNA if antioxidants do not neutralise them. However, the production of RONS is not inherently harmful; a moderate increase is crucial for cellular signalling and athletes' adaptation processes. This idea is rooted in the concept of hormesis, which describes that low levels of oxidative stress can trigger adaptive biological responses and stimulate processes that enhance cellular resilience and health [8]. Engaging in excessive or uncontrolled physical activity can lead to chronic oxidative stress, which impairs cellular function and increases the risk of inflammation-related diseases, including overtraining syndrome. Without adequate recovery, overtraining can deplete antioxidant reserves, resulting in muscle fatigue, immune suppression, and diminished performance [9]. It is important to balance the intensity and duration of exercise to prevent excessive oxidative stress while still gaining the benefits of training. Diet and supplementation play an important role in managing oxidative stress caused by exercise. Nutrients such as vitamins C and E, polyphenols, and other antioxidants found in fruits and vegetables help reduce RONS harmful activity.

## Discussion

Antioxidant enzymes are essential for the body's defence against oxidative stress, helping to maintain cellular homeostasis by regulating RONS optimal level. The three main antioxidant enzymes are superoxide dismutase (SOD), catalase (CAT), and glutathione peroxidase (GPx) [9].

**Figure 1:**
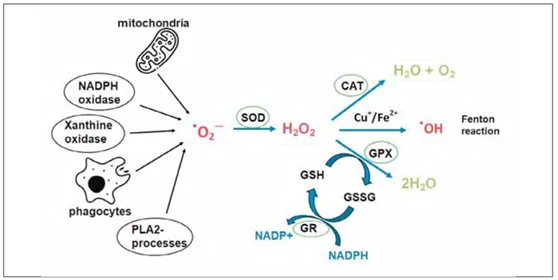
Antioxidant Enzyme Pathway: Reaction Mechanisms of SOD, CAT, GPx, and GR.<br>SOD, superoxide dismutase; CAT, catalase; GPx, glutathione peroxidase; GR, glutathione reductase; GSH, reduced glutathione; GSSG, oxidised glutathione; PLA2, phospholipase A_2_.

Superoxide dismutase exists in three isoforms: cytosolic copper-zinc SOD (Cu/Zn SOD; SOD1), mitochondrial manganese SOD (Mn SOD; SOD2), and extracellular copper-zinc SOD (Cu/Zn SOD; SOD3) [10]. These isoforms convert the superoxide anion into hydrogen peroxide (H_2_O_2_). Although H_2_O_2_ is less reactive than O_2_'^-^, it can generate hydroxyl radicals in the presence of transition metals, through the Fenton reaction, which are among the most reactive free radicals [11]. To neutralise H_2_O_2_, catalase and glutathione peroxidase convert it into water and oxygen. Additionally, important enzymes such as glutathione reductase (GR) help maintain redox balance by converting oxidised glutathione (GSSG) back to its reduced form (GSH), which is essential for glutathione peroxidase activity (Figure 1). Other contributors to redox homeostasis include peroxiredoxins, thioredoxin reductase, and heme oxygenase-1, each playing unique roles in preserving redox status [12].

### Effects of antioxidant supplementation in the athlete population - non-enzymatic antioxidants

In recent years, antioxidant supplementation has become popular among athletes looking to improve their diet, optimise recovery, and manage exercise-induced oxidative stress. Many believe these supplements can help reduce fatigue, lessen muscle damage, and boost immunity, leading to better sports performance. Commonly used non-enzymatic antioxidants include vitamins C and E, certain minerals, and polyphenols. However, studies on the effectiveness of these supplements yield mixed results, likely due to differences in study design, duration, athlete type, exercise intensity, and the specific supplements assessed.

Vitamin C is recognised for its ability to scavenge RONS, regenerate vitamin E, and support immune function. While some studies indicate that vitamin C can enhance muscle strength and reduce oxidative damage [13][14], others suggest it may impair training adaptations [15][16]. Vitamin E helps protect cell membranes from lipid peroxidation and has been shown to protect against oxidative damage and inflammation [17][18], but excessive use may blunt training benefits [19] (Table 1).

**Table 1:** Detailed information on single nucleotide polymorphisms (SNPs) related to leakage factors and outcome factors. TAS, total antioxidant status; CK, creatine kinase; LDH, lactate dehydrogenase; S100B, S100 calcium-binding protein B; MDA, malon-dialdehyde; CP carbonylated proteins; hs-CRP high sensitive C-reactive protein; FRAP ferric reducing ability of plasma; IL-1ra, interleukin 1 receptor antagonist; SOD, superoxide dismutase; ↑, increased; ↓, decreased; ↔, unchanged.

Antioxidant	Participants	Exercise	Study duration	Supplementation	Significant outcomes	Ref.
Vitamin C	9 people naive to resistance exercise	series of push-pull repetitions, including a maximal effort set, before and after the supplementation period	28 days	Vitamin C 250 mg/12 h	↑peak muscular pushing force; ↓exercise-induced oxidative stress (MDA)	[13]
Vitamin C	15 recreationally active males; EXP gr (n=8); CON. gr (n=7)	high-intensity interval running protocol, 4 times per week	4 weeks	Vitamin C 1g/day	↔training-induced improvements in exercise performance	[14]
Vitamin C	24 healthy males and females;EXP gr. (n = 12); CON. gr. (n = 12)	Anaerobic: 40 (2 x 20) maximal eccentric contractions of the elbow flexors	8 days (3 days prior to an exercise bout and 5 days after)	Vitamin C 3 g/day (3x1000 mg)	↔delayed onset muscle soreness ↔muscle strength	[15]
Vitamin C	19 healthy women; placebo-controlled crossover design	30 min moderate-intensity cycling	One dose prior to an exercise bout	Vitamin C 1000 mg	↓MDA↑ FRAP↔SOD↔CK↔LDH↔hs-CRP	[16]
Vitamin E	9 Healthy, physically active males, crossover design	60 min of exercise (70% maximal oxygen uptake) on a treadmill under normal conditions and hypoxic conditions	One hour before exercise in hypoxic conditions	Vitamin E 250 mg	↓CK total ↓ CK-MB↓LDH↓IL-1ra	[17]
Vitamin E	10 malebasketballplayers	Stretching, technical-tactical part, a heavy training load part (90 min.)	30 days	Vitamin E 200 mg/day	↓CK↓LDH ↓S100B↑TAS	[18]
Vitamin E	20 male students	incremental exercise test	8 weeks	Vitamin E 450 mg/day	↔CK↔MDA↔CP	[19]

Polyphenols, like quercetin, are known for their strong antioxidant properties. They have been associated with reduced oxidative stress and performance gains in some studies, but results vary across different athletic populations [20][21]. N-acetyl cysteine (NAC) supports glutathione level, enhances antioxidant defences, and reduces inflammatory cytokine responses in athletes [22], but its effectiveness depends on dosage and duration of supplementation. The effect can be blunted if only one high dose is used in well-trained athletes [23]. Astaxanthin (Asx) is a xanthophyll, a red-orange carotenoid found in marine organisms, primarily synthesised by microalgae and macroalgae. Asx exhibits stronger biological and antioxidant activities than other carotenoids due to its unique structure and ability to act within cell membranes [24]. Generally, Asx improves antioxidant status and may enhance recovery and reduce post-exercise immune activity decrease, especially at higher doses or in endurance-focused protocols [24][25] (Table 2). There is evidence that excessive antioxidant use may hinder training adaptations, so supplementation should be approached with caution [26].

**Table 2:** Summary of selected research studies concerning the effect of non-vitamin antioxidant supplementation on exercise-related oxidative stress. TAS, total antioxidant status; CK, creatine kinase; LDH, lactate dehydrogenase; MDA, malondialdehyde; CP carbonylated proteins; hs-CRP high sensitive C-reactive protein; GSG, glutathione; GSSG, oxidised glutathione; TBARS, thiobarbituric acid-reactive substances; XO, xanthine oxidase; NF-κB, nuclear factor k B; TAC, total antioxidant capacity; IL-6, interleukin 6; NAC, N-acetyl cysteine; SOD, superoxide dismutase; sIgA, salivary immunoglobulin A; AST, aspartate aminotransferase; ↑, increased; ↓, decreased; ↔, unchanged.

Antioxidant	Participants	Exercise	Study duration	Supplementation	Significant outcomes	Ref.
Quercetin	12 regularly active/fit men and women, placebo-controlled crossover design	Bicycle ride at 75% VO_2max_ to fatigue before and after supplementation	7 days	Quercetin 1000 mg/day	↑Time to fatigue at a constant power outputof 75% VO_2max_	[20]
Quercetin, Vitamin D	35 participants (Placebo n = 12; vitamin D (D) n = 11; Quercetin (Q) n=6; Q + D n=6)	5-km running time trial	8 weeks	Quercetin 1000 mg/day;vitamin D3 4000 IU/day	↓CP(Q+D)↓SOD(Q + D)↔ exercise performance (Q, D, Q+D)	[21]
NAC	11 male well-trained cyclists,placebo-controlled crossover design	Two maximal efforts test of a ~ 4-min duration on the bike ergometer, separated by 90 minutes	60 min before the test	NAC 20 mg/kg	↔Peak Powers ↔Blood Lactate↔CyclingEconomy↔VO_2max_↔TAC	[22]
NAC	8 Well-trained triathletes, placebo-controlled crossover design	cycle ergometer race simulation on bike ergometer	9 days and 2 hbefore the test Washout period: 21 days	NAC 1200 mg/day	↑average power 5, 10, 15 s↔total work↔blood lactate↑TAC ↑GSH ↔ GSSG↔XO↓TRABS ↔FRAP↓IL-6	[23]
Astaxanthin	40 trained male soccer players EXP. gr (n = 21); placebo (n = 19)	5 to 7 usual training sessions per week, with an average weekly training of 10 to 15 hours	90 days	Astaxanthin 4 mg/day	↑sIgAresponse ↓ AST ↓ LDH ↓ hs-CRP	[24]
Astaxanthin	18 Healthy male and female runners; placebo- controlled crossover design	running 2.25 h at close to 70%VO_2max_ after supplementation period	4 weeks	Astaxanthin8mg/day	↔muscle soreness/damage↓ post-exercise decrease in immune-related plasma proteins	[25]

### Effects of antioxidant supplementation in the athlete population - enzymatic antioxidants

**Figure 2:**
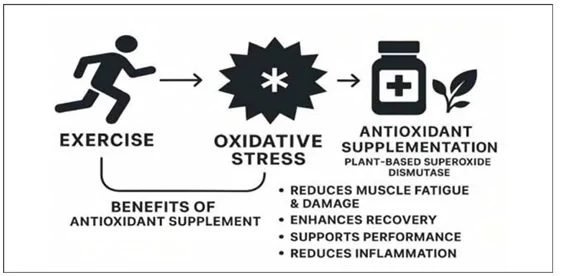
Schematic picture of the relation between exercise, oxidative stress and antioxidant supplementation.

Research on antioxidant supplements increasingly highlights the regenerative properties and effectiveness of plant-derived enzymatic antioxidants in reducing exercise-induced oxidative stress. These enzymes neutralise free radicals repeatedly, making their supplementation a promising strategy for improving athlete recovery and performance (Figure 2). This review assesses the efficacy of plant-based enzymatic antioxidants across various athletic populations to determine their benefits [26].

The main challenge with enzymatic antioxidants in supplements is their bioavailability. As proteins, enzymes can be denatured in the acidic, proteolytic environment of the gastrointestinal tract, losing their antioxidant properties. To address this, gastro-resistant formulations are essential. Strategies include the use of enteric coatings to protect macromolecules and permeation enhancers to improve absorption [27]. One of the earliest successful approaches has involved lipid-based encapsulation, which protects bovine SOD and significantly increases its bioavailability [28].

Additionally, a high-activity SOD extract from cantaloupe melon (*Cucumis melo L*.) coated with palm oil in microgranule form has shown promising results [29]. Sudareva et al. [30] conducted an in vitro evaluation of an SOD oral delivery system using different calcium alginate granules produced by different methods. SOD activity was preserved after treatment in simulated gastric acidic media, and release of the active ingredient was slow in alkaline intestinal fluid [30]. The most studied formulation is Glisodin (Isocell Nutra, Paris, France) (Table 3). It combines standardised cantaloupe protein extract (average SOD 100 IU/mg of dry extract) with wheat (Triticum vulgare) gliadin biopolymers in a specific ratio to achieve a final activity of 1 IU SOD/mg in the final dry powder [31]. Gliadin provides gastroresistant properties and enhances delivery through the digestive system [32]. Gliadin serves as an effective gastroresistant carrier for superoxide dismutase (SOD), protecting the enzyme from degradation in the stomach's acidic environment and from digestive enzymes. This combination allows SOD to remain active until it reaches the intestine for better absorption. In vitro studies mimicking digestion demonstrate that SOD activity is preserved progressively with gliadin, unlike unprotected SOD, which rapidly loses function. Gliadin promotes intestinal permeability by triggering zonulin release, facilitating SOD transport across the gut barrier into the bloodstream. Its bioadhesive qualities further aid SOD diffusion into the intestinal mucosa [32][33].

**Table 3:** Glisodin ingredients.

Usual Name	Latin Binomial	Plant Part	CAS #
Melon<br>concentrate	*Cucumis melo* L.	Fruit pulp	90063-94-8
Gliadin	*Triticumvulgare*	Wheat grain	9007-90-3
Maltodextrin	*Triticum* spp.	Wheat grain	9050-36-6

In a proof-of-concept study, gliadin-coated SOD supplementation in mice improved antioxidant enzyme levels significantly compared to uncoated SOD and placebo [31]. Other studies have shown benefits in red blood cells' resistance to oxidative stress and increased hepatocyte resilience against peroxynitrite-induced apoptosis [34].

The health benefits of orally administered SOD/gliadin have been examined in various fields, including metabolic disorders, cardiovascular diseases, inflammation, cancer, infections, brain function and sports nutrition [35][36]. Research involving CnZn-SOD knockout mice has also been conducted to understand better understand the role of SOD in maintaining oxidative balance during physical activity. Insights from knockout mouse models have generated hypotheses about the potential use of SOD supplementation in athletes.

### Plant-based superoxide dismutase in sport supplementation

Elite athletes engage in regular, high-intensity training with limited recovery periods, making them especially vulnerable to exercise-induced oxidative stress. In the ongoing search for an optimal antioxidant that can attenuate the detrimental effects of oxidative stress without hindering physiological adaptation, plant-based enzymatic antioxidants, most notably superoxide dismutase, have emerged as a promising area of investigation [37].

A limited number of studies have investigated antioxidant supplementation in specific groups, including rowers [38][39][40], soccer players [41], and a healthy population that does not train regularly [42]. One study included professional divers, but it was excluded from this analysis because oxidative stress was induced by exposure to pure oxygen in a hyperbaric chamber for 60 minutes, not by physical exercise [43]. All studies reviewed here focus specifically on oxidative stress resulting from exercise and athletic activity (Table 4).

**Table 4:** Comparative overview of studies on SOD/gliadin dietary intake effects on exercise-related oxidative stress. EXP gr, experimental group; CON gr., control group; TAS, total antioxidant status; TOS, total oxidant status; OSI, oxidative stress index; SOD, superoxide dismutase; GPx, glutathione peroxidase; GR, glutathione reductase; LPO, lipid peroxides; 8-iso PGF2α, 8-isprostane; TBARS, thiobarbituric-acid-reactive substances; AOPP advanced oxidation protein products; MDA, malondialdehyde; CK, creatine kinese; LDH, lactate dehydrogenase; CRP C reactive protein; IL, 6-interleukin 6; Lac, serum lactate concentration; VLT, velocity at lactate threshold; La peak, the highest measured lactate concentration; W, work; Met. Eff., metabolic efficiency.

Tested<br>population	Dietary<br>intervention	Performance<br>test	Measured<br>redox<br>parameters	Measured <br>inflammation and <br>muscle damage <br>parameters	Measured<br>performance<br>parameters	Significant<br>outcomes	Ref.
44 healthy volunteer, severe exercise group (SEG n = 27); moderate exercise group (MEG n = 17)	1500 IU oral SOD(1500 mg Glisodin; 3x500 mg,4 weeks	Cycling or treadmill exercise up to 200 kcal/ 20 minutes or 300 kcal/30 minutes	TAS; SOD; GPx; GR	none	Lac	SEG exercise induced changes: ↓ΔTAS (*p* <0.05; ↓ΔSOD (*p* <0.05; ↓ΔLac(*p* <0.01)MEG: ↑ΔLac (p<0.01)	[42]
22 male division of college soccer players; EXP gr. (n = 12);CONgr. (n = 10)	Resugrex plus (combined nutriceutical blend containing 500 mg Glisodin,20 days	Graded maximal treadmill test to exhaustion	LPO; 8-iso PGF2α;	CK	V_LT_; O_2max_;time-to-exhaustion	↓baseline CK (*p* =0.044); ↓Δ8-iso PGF2α (*p* =0.004)	[41]
19 rowers of the Polish national team, EXP group.(n = 10); CON. gr. (n = 9)	500 mg Glisodin (2x250 mg), 6 weeks	2000 m maximum effort test on rowing ergometer	SOD;GPx;TBARS;TAS	CK; LDH; CRP	Lac	↑SOD(*p* =0.0037); ↓CRP (*p* <0.001)	[39]
28 international-level rowers;EXP gr. (n = 15); CON gr. (n = 13)	500 mg Glisodin (2x250 mg),6 weeks	Increased interval exercise test on the rowing ergometer	TAC;	CK; LDH; IL-6; CRP	La peak; Wmax; W at 4 mmol/L La	↓baseline CK (*p* =0.049); ↓baseline IL-6 (*p* =0.035); .↓exercise induced increase in IL-6 (*p* =0.05)	[38]
30 international-level rowers;EXP gr. (n = 15); CON gr. (n = 15)	500 mg Glisodin (2x250 mg),6 weeks	Maximal effort incremental test on the rowingergometer test	TAS; TOS; OSI; SOD; GPx; AOPP; MDA;SH group	none	Met. Eff. at max power; Met. Eff. at 15 mmol/L La; Met. Eff. at 4 mmol/L	↓TOS(*p* =0.010);↓OSI (*p* =0.004); ↓MDA (*p* =0.001);↑SH group (p=0.031); ↑Δ Met. Eff. at 4 mmol/L (*p* =0.004); ↑Δ Met. Eff. at max power (*p* =0.015)	[40]

### Effect of plant-based superoxide dismutase supplementation on redox status and oxidative stress parameters

The effects of antioxidant enzyme supplementation on redox status were examined by assessing changes in the activity or concentration of antioxidant enzymes, including SOD, GPx, CAT, and GR. General parameters of redox status, including total oxidative status (TOS) and total antioxidant status (TAS) or total antioxidant capacity (TAC), were also monitored to assess the overall oxidative balance in the subjects. Additionally, several biochemical markers of lipid oxidation, including thiobarbituric acid-reactive substances (TBARS), malondialdehyde (MDA), 8-isoprostane, and lipid peroxides (LPO), were measured, as were markers of protein oxidation, such as sulfhydryl groups (SH) and advanced oxidation protein products (AOPP). These indices provide a detailed assessment of oxidative modifications affecting both lipids and proteins during exercise and help quantify the degree of oxidative stress in athletes.

A study involving 44 healthy volunteers explored the effects of orally administered plant-based SOD (Glisodin) at a dose of 1500 IU for 4 weeks [42]. The researchers measured changes in TAS, SOD, GPx and GR levels. At the start of the study, each participant engaged in strenuous cycling or treadmill exercise, aiming to burn 200 calories in 20 minutes or 300 calories in 30 minutes, depending on their fitness level. Blood samples were collected shortly before and after intense physical activity. Participants were classified into two groups based on their blood lactate levels after exercise: those with moderate exercise (n=17) who had lactate levels less than 4.5 mmol/L, and those with severe exercise levels (n=27) who had lactate levels 4.5 mmol/L or higher. After four weeks of oral Glisodin administration among severe exercise participants, exercise-induced increases in redox parameters were minimised, as shown by reduced TAS (ΔTAS = -0.05±0.10 mmol/L, *p*<0.05) and SOD (ΔSOD=-85.2±195.5 U/g Hb, *p*<0.05). These findings indicate that Glisodin supplementation may help attenuate oxidative stress levels associated with intense exercise [44].

In a study involving 22 college soccer players, participants were supplemented with a formula containing the following ingredients: 75 mg of coenzyme Q10, 500 IU of SOD/gliadin, 1750 mg of ornithine ketoglutarate, 300 mg of L-carnitine, 100 mg of nucleotides, 750 mg of D-ribose, 500 mg of L-glutamine, 100 mg of beta-glucans, 12.5 mg of fruit polyphenols, and 1750 mg of branched-chain amino acids (BCAAs) from a product commercially named Resurgex Plus® [41]. The athletes were randomly assigned to receive either the supplement in drink form (experimental group; n=12) or an isocaloric placebo (control group; n=10) during a 20-day pre-season training period in a blinded, placebo-controlled design. At the beginning and end of the study, they underwent a progressive treadmill test to evaluate VO_2_max, velocity at lactate threshold (VLT), time-to-exhaustion, lipid peroxidation (LPO), 8-isoprostane, and creatine kinase (CK) levels. Blood samples were collected before and after each test. The results showed significant changes in oxidative stress parameters, particularly in the Group X Trial interaction for 8-iso PGF2 (*p* = 0.004). There was a significant decrease in the 8-iso PGF2 response in the supplemented group during Trial 2 (Δ8-iso PGF2 =8.2±16.3 pg/mL) compared to Trial 1 (Δ8-iso PGF2 =12.6±17.0 pg/mL), with an effect size (ES) of -0.74. Additionally, the Group X Trial interaction for LPO approached significance (*p* = 0.067), with a slight decrease in LPO response during testing at Trial 2 (ΔLPO = 1.31±0.7 μmol•L- 1) compared with Trial 1 (ΔLPO = 1.52±0.7 μmol•L-1), ES = -0.27 in the experimental group.

The study, involving 19 members of the Polish rowing team [39], used supplementation over a period of six weeks, spanning from the preparation season to the competition season. The study was a double-blinded, randomised, placebo-controlled trial. Rowers were randomly assigned to one of two groups: the supplemented group (n = 10), which received two capsules (2x250 mg (IU)) of plant extract rich in SOD combined with gliadin (Glisodin) once a day, or the placebo group (n = 9). All athletes performed a 2,000-meter maximum-effort test on a rowing ergometer before and after the supplementation period. Blood samples were collected at three time points: before each test, one minute, and 24 hours post-exercise. Changes in redox parameters were monitored by measuring SOD and GPx activity and TBARS concentration in erythrocytes.

Additionally, TAC was assessed in plasma samples. An increase in GPx activity and TAC after exercise was observed in both groups, regardless of supplementation. Nevertheless, SOD activity was significantly higher in the supplemented group before, 1 minute, and 24 hours after intensive exercise (*p* = 0.0037), which the authors considered a positive effect of supplementation.

Another randomised, double-blind, placebo-controlled study included 28 international-level rowers [38]. All participants were tested on a rowing ergometer using an increased interval step test protocol until exhaustion. The effect of supplementation on redox status was assessed using only changes in total antioxidant capacity (TAC). Supplementation lasted 6 weeks, during which the experimental group (n = 15) received 500 mg Glisodin per day (2 x 250 mg). The control group (n = 13) received placebo capsules. There were no significant changes in TAC values attributed to supplementation. Since TAC primarily reflects the presence of non-enzymatic antioxidants in plasma, this outcome is expected. It highlights a limitation of using TAC alone to assess the impact of enzymatic antioxidant supplements.

Further study involved 30 elite rowers [40]. The supplementation period was set at 6 weeks, with an incremental step test to exhaustion on a rowing ergometer at the beginning and end of the study. The experimental group (n = 15) received 500 mg of Glisodin (2x250 mg) orally once daily, 1 h before training or 1 h before breakfast, while the control group (n = 15) received placebo capsules. To assess the effects of antioxidant supplementation on redox status, a variety of parameters were measured: TOS, TAS, and the TOS/TAS ratio, known as the Oxidative Stress Index (OSI). Additionally, changes in the levels of antioxidant enzymes, such as SOD and GPx, were monitored, along with markers of lipid (MDA) and protein oxidation (AOPP and SH-groups). Supplementation with an extract rich in SOD resulted in significantly lower TOS (*p* = 0.01) and OSI (*p* = 0.004) in the experimental group. Similar to a study conducted with Polish rowers [39], the supplementation increased SOD levels, despite using different measurement methods: SOD activity in erythrocytes (39) or extracellular SOD concentration [40]. This was an expected result, aside from the fact that the resorption and mechanism of action for this enzyme are still not completely explained. However, in both studies, supplementation did not significantly affect GPx levels. The effect on protein oxidation levels was observed only at higher SH-group values, indicating lower protein oxidation in the experimental group after supplementation. A significantly lower MDA level was noted in the experimental group both before and after exercise at the end of the study, indicating lower lipid oxidation due to supplementation [40].

### Effects of plant-based SOD supplementation on inflammation and muscle damage parameters

Regular, moderate physical activity is crucial for maintaining good health and promoting longevity at any age. Exercise initiates metabolic changes and adaptive mechanisms that help establish a new balance, providing benefits related to oxidative stress and inflammation. However, prolonged, intense training without adequate rest can lead to muscle injuries, inflammation, accumulated fatigue, and disruptions in endocrine function, increasing systemic inflammation and health risks [44].

The effects of acute exercise on inflammation can vary based on factors such as the type, intensity, and duration of exercise, as well as the athlete's age and physical condition [44]. Changes are often monitored through peripheral blood cell counts, granulocyte activity, NK cell cytotoxicity, lymphocyte proliferation, cytokine levels, and positive acute-phase proteins [45]. Cytokines are signalling molecules that regulate inflammation and immune responses. During inflammation, TNF- and IL-1 levels rise initially, but during exercise, IL-6 becomes the dominant cytokine, followed by anti-inflammatory cytokines such as IL-10 and the IL-1 receptor antagonist (IL-1ra) [46]. IL-6 also triggers the release of C-reactive protein (CRP), a hepatic acute-phase protein that serves as a marker for systemic inflammation. Some research has indicated a moderate rise in CRP levels after intense exercise [46][47][48]. Still, results vary, with discrepancies noted in the timing and extent of CRP elevation, whether immediately after exercise or 24 hours later.

Oxidative stress can activate various transcription factors, including NF-κB, Peroxisome Proliferator-Activated Receptor gamma and beta (PPAR-γ, β), Nuclear factor (erythroid-derived 2)-like 2 (Nrf2), and Activator Protein-1 (AP-1). This activation leads to the expression of over 500 genes, including those encoding growth factors, inflammatory cytokines, chemokines, cell cycle regulatory molecules, and anti-inflammatory molecules [49]. To assess the effects of antioxidant supplementation on inflammation levels in athletes, researchers focused on CRP and IL-6.

Intense, prolonged exercise can cause muscle tissue injury due to both metabolic and mechanical factors. Serum levels of skeletal muscle enzymes such as creatine kinase (CK), lactate dehydrogenase (LDH), aldolase, myoglobin, troponin, and aspartate aminotransferase (AST) serve as markers of muscle function and can fluctuate significantly under both physiological and pathological conditions. Muscle injuries following strenuous exercise can be exacerbated by increased oxidative stress. Elevated serum levels of the muscle enzyme CK are widely recognised as indicators of tissue damage [50]. CK is involved in muscle energy metabolism, and its blood concentration can temporarily rise after strenuous exercise or heavy physical activity due to increased muscle cell permeability. The degree of CK elevation varies based on the type and duration of exercise, with untrained individuals typically experiencing a greater increase. Exercise also induces a significant rise in LDH. The body contains five isoenzymes of LDH, each contributing to the conversion of pyruvate to lactate and favouring either aerobic or anaerobic metabolism, depending on the specific isoenzyme's structure. The overall increase in LDH following exercise reflects total LDH levels, while individual isoenzymatic fractions may remain unchanged. These changes depend on the intensity and duration of the exercise effort [51].

To investigate the possible effects of SOD supplementation on inflammation and muscle damage, the researchers monitored several parameters. In a study with a college soccer team, 20 days of supplementation resulted in a significantly lower resting CK level in the experimental group compared to the control group (*p*=0.044) [41]. In particular, the experimental group showed a smaller increase in resting CK levels at the end of the study (ΔCK=64.8±188.4 U/L), whereas the control group showed a larger increase (ΔCK=292.8±304.8 U/L) compared to the initial level before supplementation. After exercise, CK levels were elevated, and no differences were observed between the groups. It is important to note that the blood samples were taken 5 minutes before and after exercise. Accurate changes in this parameter would be measured by tracking CK levels over a longer period, such as 24 hours; however, this did not fit within the scope of the study.

In the study with the Polish rowing team, six weeks of supplementation showed no significant effect on the muscle damage parameters CK and LDH. Physical activity increased both enzymes, but no differences were found between groups. However, the supplemented group showed significantly lower CRP values (*p*<0.001) at all three measurement points: before the test, one minute after, and 24 hours after the ergometer test. Some studies suggest that the damage to muscle cells caused by free radicals generated during exercise can trigger inflammation [52]. The observed reduction in CRP levels was not associated with decreased muscle damage parameters, suggesting that the anti-inflammatory effect was not correlated with muscle damage.

In another study investigating the effects of Glisodin supplementation among 28 international-level rowers, a significantly lower resting CK value was found in the supplemented group (*p* = 0.049) [38]. This result mirrors findings in soccer players [41] and suggests that supplementation can improve muscle recovery after intensive exercise. However, supplementation did not significantly affect CRP levels in this study. It is noteworthy that IL-6 level, an important marker of inflammation, was lower in the supplemented group both before (*p*=0.035) and after physical activity (*p* = 0.050) at the end of the study. IL-6 is a versatile cytokine that has a broad spectrum of biological effects. It is produced by contracting skeletal muscle during prolonged exercise [53]. With adequate rest and proper nutrition, the IL-6 response to exercise decreases as skeletal muscle adapts. Persistently high IL-6 levels can interfere with the body's normal adaptive responses to exercise [54]. Therefore, elite athletes need to keep IL-6 levels within a controlled range, which could have a protective effect on muscles. Taking Glisodin could help to maintain this IL-6 level and thus make an essential contribution to keeping athletes in good shape.

### Effects of plant-based SOD supplementation on athletes' performance

The various metabolic and physiological changes that professional athletes experience as a result of frequent and intense training, especially when they do not have sufficient time to recover, are important for both their health and their physical performance. Oxidative stress induced by exercise is associated with muscle damage, lipid peroxidation, fatigue, and slower recovery, all of which can negatively impact athletes' performance.

VO_2_max is a key parameter used to measure physical ability, particularly in endurance sports. Defined as an athlete's aerobic capacity, VO_2_max was first introduced by Hill and Lupton in 1923. It reflects the maximum ability of the muscles, lungs, and circulatory system to absorb, deliver, and utilise oxygen during prolonged, intense exercise [55]. However, it is important to note that VO_2_max can change very little with short-term training, especially in elite athletes, who have most likely reached their full potential. While improvements are possible, they are limited by the cardiopulmonary system's ability to supply oxygen to the muscles and are also influenced by genetic factors [56].

In contrast, lactate levels are believed to correlate much better with an athlete's performance. This strong correlation arises because lactate not only indicates how effectively oxygen is delivered to and utilised by the muscles, but also reflects the muscles' metabolic state and the rates of aerobic and anaerobic energy processes [57][58]. Hence, in the studies analysed, lactate levels were considered an important parameter for assessing athletes' physical abilities.

In a study involving 44 healthy non-athlete volunteers, lactate levels were measured at the beginning, after initial testing on a cycling or treadmill machine [42]. Based on the test results, participants were divided into two groups: those with moderate exercise and those with severe exercise levels. After four weeks of supplementation with 1500 IU of SOD (1500 mg of Glisodin), the severe exercise experimental group showed a significantly lower exercise- induced lactate levels (ΔLac=-3.9±4.1 mmol/L, *p*<0.01). In contrast, the moderate exercise group experienced a significant increase in exercise-induced lactate levels change (Δ Lac=1.4±1.7 mmol/L, *p*<0.01). One possible explanation for these results is that the moderate exercise group did not reach an intensity sufficient to induce anaerobic metabolism; therefore, their increase in lactate levels was minimal and aligned with typical metabolic changes associated with exercise. Consequently, antioxidant supplementation did not further reduce lactate levels in this group. There is a positive correlation between SOD supplementation and changes in lactate levels (r=0.76, *p*<0.01). This indicates that a stronger effect of SOD supplementation can be expected with a higher initial rise in exercise-induced lactate.

In a study examining the effects of supplementation in a group of soccer players, sports performance was assessed through: Velocity at Lactate Threshold (VLT), maximum oxygen uptake (O_2_max), and total time spent running during each test, which was used to determine total time to fatigue for each participant [41]. The researchers plotted lactate concentration against treadmill speed to determine VLT and measured O_2_max using direct breath-by-breath gas-exchange data. Throughout the study, both the experimental and control groups showed improvements in all parameters, though there were no significant differences between the two groups. However, the estimated effect sizes (ES) for supplementation indicated a positive trend in the experimental group compared to the control group. Specifically, the effect sizes in the experimental vs. control group were as follows: VLT ES = 0.93 vs. 0.44; O_2_max ES=0.57 vs. 0.34; and time to exhaustion ES=0.51 vs. 0.26. The researchers concluded that, despite the short supplementation period, the positive shifts in the measured biochemical parameters suggest that antioxidant supplementation may enhance athletic performance. In a separate study involving a Polish group, changes in rowers' performance were not monitored [39].

In a study involving 28 international-level rowers, researchers assessed the impact of supplementation on performance by monitoring changes in lactate concentration and the amount of work performed (measured in watts) during a rowing ergometer test with increasing load until exhaustion [38]. They calculated the amount of work completed by the athletes at a specific lactate concentration of 4 mmol/L, which represents the metabolic efficiency at this level (W at 4 mmol/L La). This lactate concentration is widely recognised as the point at which blood lactate accumulation is likely to begin, known as the onset of blood lactate accumulation (OBLA). Additionally, the maximum lactate level (La peak) measured immediately after completing the ergometer test was compared, along with the maximum work achieved (W max) during rowing until exhaustion. After six weeks of supplementation, there were no statistically significant changes in La peak or W max. However, the rowing power at the 4 mmol/L lactate concentration post-supplementation was significantly higher in the experimental group (ΔW at 4 mmol/L La, *p* = 0.020), indicating improved metabolic adaptation to intensive exercise.

A study involving 30 international-level rowers investigated the effects of supplementation on metabolic efficiency, specifically by measuring the power-to-lactate ratio at 4 mmol/L and 15 mmol/L, and maximum tested power at ergometer testing [40]. The work value calculated at a lactate concentration of 15 mmol/L reflects the metabolic efficiency typically seen in rowers after a 2000 m race. The researchers evaluated changes in work performance within the groups and calculated the percentage change between initial and final testing conducted before and after supplementation. Significant differences emerged between the groups regarding changes in metabolic efficiency throughout the supplementation period. Notably, metabolic efficiency at maximum tested power was higher in the experimental group after supplementation (*p*=0.015). Additionally, metabolic efficiency at 4 mmol/L lactate was significantly greater in the supplemented group than in the control group (*p* = 0.004). Supplementation with Glisodin appeared to enhance metabolic efficiency in the experimental group during the specific ergometer test used in this study, indicating improved performance among the rowers.

These findings suggest that Glisodin could serve as an effective nutritional support during demanding physical activities, helping to mitigate the harmful effects of oxidative stress on muscle damage, inflammation, and overall athletic performance.

## Conclusion

Based on the studies included in this review, supplementation with plant-based SOD combined with gliadin shows significant potential as an effective sports supplement. This combination appears to positively influence athletes' redox status by enhancing antioxidant defences, thereby reducing exercise-induced oxidative stress and inflammation. These biochemical benefits translate into practical outcomes such as improved muscle recovery and reduced inflammatory markers, which are critical factors affecting training adaptation and performance longevity.

Importantly, the evidence reviewed suggests that Glisodin supplementation may enhance performance parameters in sports that require a combination of endurance, strength, and recovery, such as soccer and rowing. These findings highlight the potential of plant-based SOD combined with gliadin as a novel, natural nutritional strategy to support both athlete resilience and performance optimisation.

While promising, these conclusions should be interpreted with caution given certain limitations in the current body of research. Many studies feature small sample sizes, varied supplementation protocols, and short intervention durations, which limit the generalizability and long-term applicability of their findings. Additionally, the mechanisms by which gliadin enhances the bioavailability and efficacy of orally administered SOD remain relatively unexplored, indicating a need for further biochemical and pharmacokinetic analyses.

To strengthen the evidence base, future research must prioritise large-scale, randomised controlled trials with standardised dosing regimens and diverse athlete populations. Longitudinal studies that assess not only immediate recovery effects but also sustained performance outcomes over entire seasons would be particularly valuable for gaining insight into possible effects on athletes' adaptive mechanisms. Furthermore, exploring potential synergies between plant-based SOD supplementation and other nutritional or training interventions could uncover optimised strategies for athlete health management.

Overall, plant-based SOD combined with gliadin emerges as a promising natural antioxidant intervention with meaningful implications for sports nutrition. Its capacity to mitigate oxidative stress and inflammation, coupled with performance benefits, positions this supplementation as a valuable adjunct to conventional training and recovery protocols. Continued investigation and validation of these findings have the potential to advance practical recommendations and enrich the toolkit available to athletes and sports health professionals striving for enhanced performance and well-being.

## Dodatak

### Conflict of interest statement

All the authors declare that they have no conflict of interest in this work.
